# Larval diet-mediated morphological trait and *Wolbachia* density variation in *Wolbachia*-transinfected (*w*Mel and *w*AlbB) and *Wolbachia*-free *Aedes aegypti* mosquitoes

**DOI:** 10.3389/finsc.2026.1887426

**Published:** 2026-09-03

**Authors:** Aravind Revi, Yazhini Gunasekaran, Vidhya Pachalil Thiruvoth, Amala Ramasamy, Waseema Arif, Manimaran Kuppusamy, Devi Venugopal, Kayalvizhi Ebinezar, Esther Bakianathan, Alosia Essoudasse, Pavithra Parandamane, Amrutha Vijayan, Tamilselvan Anbazhagan, Esther Nirmala Mary, Hemasankar Ramkumar, Ananganallur Nagarajan Shriram, Manju Rahi

**Affiliations:** Indian Council of Medical Research-National Institute for Vector Control Research, Department of Health Research, Ministry of Health & Family Welfare, Government Of India, Medical Complex, Puducherry, India

**Keywords:** *Aedes aegypti*, fitness characteristics, larval diet, *w*AlbB, *w*Mel, *Wolbachia*

## Abstract

**Introduction:**

Larval diet plays a crucial role in mosquito development and fitness by influencing both larval and adult traits. During the aquatic stage, mosquitoes acquire essential nutrients required for successful metamorphosis and adult emergence. Interactions between larval nutrition and bacterial endosymbionts such as *Wolbachia* further influence mosquito development, with important implications for field-based vector control strategies.

**Methods:**

We evaluated the effects of four larval diets on fitness characteristics of *Wolbachia*-transinfected and uninfected *Ae. aegypti* strains. The diets tested were: LD1 (fish feed), LD2 (laboratory rodent diet), LD3 (mushroom powder), and LD4 (dog biscuit plus brewer’s yeast). We assessed wing length and body weight in *Wolbachia*-transinfected strains (*w*Mel and *w*AlbB) and uninfected strains across two generations of *Ae. aegypti* reared under each larval diet regimen. Additionally, *Wolbachia* density was measured in the transinfected *Ae. aegypti* strains using real-time PCR.

**Results:**

Female wing length was significantly influenced by the interaction between diet, *Wolbachia* strain, and generation (Diet × Strain × Generation: F = 3.98, P = 0.0044**),** together with significant Diet × Strain (F = 3.05, P = 0.0135. In contrast, male wing length was primarily affected by diet (F = 14.11, P < 0.001) and the Diet × Generation interaction (F = 5.58, P = 0.0019). Male body weight also showed a significant three-way interaction among diet, strain, and generation (F = 8.95, P < 0.001), indicating strain- and generation-specific dietary responses. Female body weight exhibited significant Diet × Strain (F = 3.43, P = 0.0069 and Strain × Generation (F = 7.79, P = 0.0012) interactions. Overall, larval diet was the primary determinant of mosquito fitness, with its effects modified by *Wolbachia* infection status and generation.

**Discussion:**

This study emphasizes that larval diet significantly influences the fitness of both *Wolbachia-*transinfected and uninfected *Aedes aegypti* strains. LD1 and LD4 showed improved wing development and fitness compared to LD2 and LD3, with sex-specific effects observed. Based on the analysis, LD1 is recommended for routine mass rearing of *Wolbachia* trans-infected mosquitoes. These findings highlight the need for future studies on cost-effective, locally available diet formulations to optimize mosquito fitness for vector control strategies.

## 1 Introduction

Arboviral infections are primarily transmitted to vertebrate hosts by blood-feeding arthropod vectors. Over 700 human-pathogenic arboviruses have been documented globally, with the majority disseminated by mosquitoes in the family Culicidae (1). Among these, Chikungunya virus (CHIKV), Dengue virus (DENV), Yellow fever virus (YFV), and Zika virus (ZIKV) represent the most significant in terms of pathogenic impact and human burden (2), all transmitted by *Aedes aegypti*. The principal vector of Dengue in India is *Aedes (Stegomyia) aegypti* (L.), with *Aedes (Stegomyia) albopictus* (Skuse) playing a secondary role in transmission (3). Dengue is the most rapidly expanding arboviral infection globally, with cases reported to the WHO escalating from 505,430 in 2000 to 14.6 million in 2024 (4, 5). Dengue, once endemic in only nine countries in 1970, has now spread to over 120 countries. In India, 121,824 confirmed dengue cases and 131 deaths were recorded in 2025, with 6927 cases and 10 deaths reported till February 2026 (6). This rising burden is driven by rapid urbanization, lifestyle changes, and poor water management practices across urban, peri-urban, and rural settings (7).

In the absence of universally efficacious vaccines and therapeutics, vector control and patient management remain the primary strategies for dengue control (8, 9). Dengue vector control strategies are primarily centered on insecticide-based conventional tools and source reduction approaches so far. However, the widespread emergence of insecticide resistance in *Ae. aegypti* populations have significantly compromised the efficacy of these approaches (10), necessitating the urgent exploration of alternative vector control methods (9). Methods such as Sterile Insect Technique (SIT) and *Wolbachia*-based vector control strategies are being explored to combat dengue and other mosquito-borne diseases (11, 12).

*Wolbachia* is a genus of Gram-negative, intracellular, maternally transmitted endosymbiotic bacteria, which was first discovered as *Rickettsia*-like organisms from the germ cells of *Culex pipiens* (13). Natural *Wolbachia* infection has been reported in more than 60% of all insect species, including mosquitoes like *Culex pipiens* and *Aedes albopictus* (14)*. Ae. aegypti*, the major vector of dengue, is generally considered to be free of natural *Wolbachia* infection (15), although occasional reports have detected the presence of *Wolbachia* in natural populations of this mosquito (16–18). The existence of stable natural infections of *Wolbachia* in *Ae. aegypti* remains a subject of ongoing debate. *Wolbachia*, when present in mosquitoes, have the potential to manipulate the reproductive biology of the host organism through a variety of mechanisms. One of these mechanisms is cytoplasmic incompatibility, in which the embryos fail to develop when the *Wolbachia*-free females are mated with *Wolbachia*-infected males (19). *Wolbachia-*infected *Ae. aegypti* can be used as either population replacement or population suppression strategies, which depend on *Wolbachia*-induced cytoplasmic incompatibility (20, 21). The population replacement strategy involves the simultaneous release of male and female mosquitoes infected with *Wolbachia* to gradually substitute the natural *Wolbachia*-free mosquito population with a *Wolbachia*-infected population. (22), whereas the population suppression approach involves releasing *Wolbachia*-infected males, which results in non-viable offspring when they mate with wild females (23). *Wolbachia* release trials conducted in numerous parts of the globe have shown a significant reduction in the number of notified dengue and chikungunya cases, notably in countries like Australia, Indonesia, Brazil, Colombia, and Malaysia (24–28). Among all the trials, the one conducted in the city of Yogyakarta, Indonesia, is marked as the gold standard trial because of its broad success ratio, which resulted in a 77% reduction in virologically confirmed dengue case incidence in *Wolbachia*-treated areas compared with untreated areas over a period of 27 months (29). Currently, field releases of *Aedes aegypti* mosquitoes infected with the *Wolbachia* strain *w*Mel are underway in 11 countries to assess their effectiveness in controlling dengue (30).

A joint venture between the Indian Council of Medical Research - National Institute for Vector Control Research (ICMR-NIVCR), India, and the World Mosquito Program from Monash University, Australia, developed two unique *Ae. aegypti* strains that carry *w*Mel and *w*AlbB *Wolbachia* strains. These strains were developed by doing multiple backcrosses between the *Wolbachia*-infected female *Ae. aegypti* (*w*Mel and *w*AlbB Australia) and uninfected wild *Ae. aegypti* Puducherry (Pud) male over six generations (31). The operationalization of *Wolbachia*-based vector control in field conditions requires large-scale rearing and release of mosquitoes with optimum fitness characteristics (32). One of the pivotal factors in mass mosquito rearing, which leads to optimum biological fitness, is the larval diet provided during the early life cycle of mosquitoes. Many studies have been conducted in different parts of the world by comparing various larval diets to know the effect of various diets on mass-reared *Ae. Aegypti*, especially for the Sterile Insect Technique (SIT) (33, 34).

A recent study conducted at ICMR-NIVCR demonstrated that larval diet significantly influenced several life table characteristics of *w*Mel and *w*AlbB Puducherry strains, such as mean hatchability rate, pupation, adult emergence, male-female ratio, fecundity, and adult survival (32). Since limited information is available on the influence of larval diets on important morphological and biological parameters, such as body weight, wing length, and *Wolbachia* density across generations, we conducted this study to comprehensively evaluate the effects of different larval diets on these characteristics. Enhanced larval nutrition often results in larger mosquitoes, and larger body size has been linked to increased fecundity, greater sperm transfer, improved survival, enhanced mating success and fitness (35, 36). According to Lounibos (37), a linear measurement of wing lengths of mosquitoes can be used as a surrogate measure of body size. Therefore, the current research determined which larval diet best supports adult morphological traits in each *Wolbachia* strains (*w*Mel and *w*AlbB *Aedes aegypti*) relative to the uninfected *Ae. aegypti*, with a focus on detecting statistically significant differences in morphological outcomes across selected diets and strain combinations. Four larval diets were assessed, including fish feed (TetraBits granules), laboratory rodent diet, mushroom powder, and the standard diet composed of dog biscuit and brewer’s yeast in a 3:2 ratio. Parameters such as body weight, wing length and *Wolbachia* density were assessed across two generations to identify a suitable larval diet for the mass rearing of *Wolbachia*-transinfected *Ae. aegypti* mosquitoes.

## 2 Materials and methods

### 2.1 Study design

**Figure 1:**
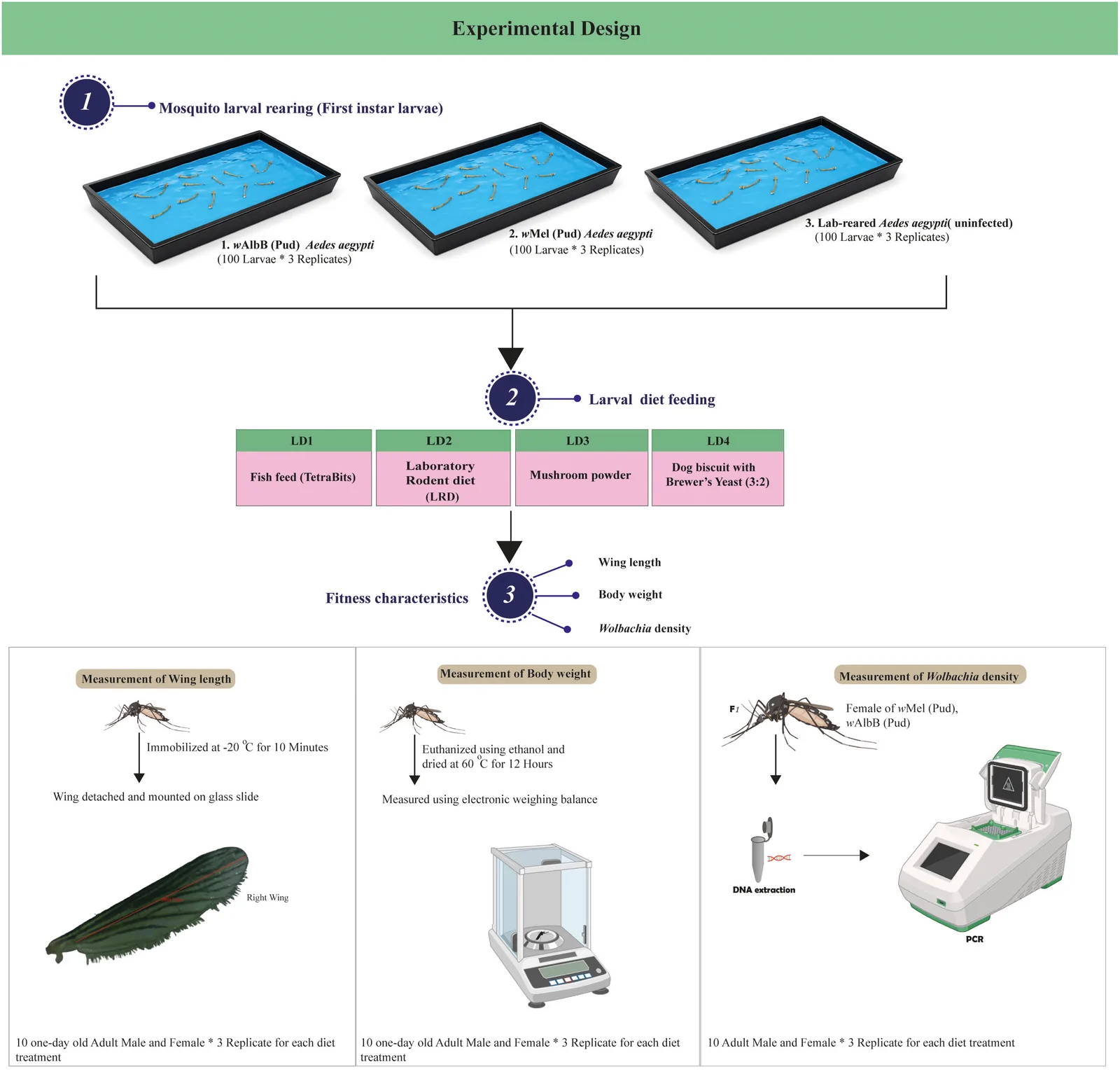
Schematic representation of the study design used to evaluate the effects of four larval diets on the fitness characteristics of three *Ae. aegypti* strains: wAlbB (Pud), wMel (Pud), and uninfected. (Created with BioRender.com)

This study employed an experimental laboratory design to evaluate the effects of larval diet, *Wolbachia* infection status, and mosquito generation on key life-history and endosymbiont traits in *Aedes aegypti*. The design comprised four larval diet regimens (LD1–LD4), three *Ae. aegypti* strains (*w*Mel-infected, *w*AlbB-infected, and *Wolbachia*-uninfected), and two generations (F0 and F1), yielding a full factorial design of 4 × 3 × 2 = 24 diet–strain–generation combinations. Each combination was assessed using three independent biological replicates, with each replicate initiated from 100 first-instar larvae (n = 300 larvae per diet–strain–generation combination; total n = 7,200 larvae across the full design). One treatment combination, LD2 (rodent diet) in the F1 generation of the *w*Mel strain, could not be completed, as no adults survived to a stage permitting assessment of wing length or body weight. All other 23 combinations were completed as planned. An overview of the study design and workflow is presented in **Figure 1**. A sample size of n = 100 larvae per replicate (three replicates per diet combination; total n = 300 per combination) was selected.

### 2.2 Mosquito strains and colony maintenance

We obtained the *Wolbachia* transinfected *Ae. aegypti* from cyclical colonies, maintained at ICMR-NIVCR (formerly VCRC). First instar larvae of *w*Mel, *w*AlbB transinfected and uninfected *Ae. aegypti* strains were used for the study. For each strain and diet combination, three biological replicates of 100 larvae (n=300) were established. Larvae were introduced within 24 hours of hatching into enamel trays (45 × 30 × 5 cm) containing 3 L of water, following World Mosquito Program rearing protocols (38. Colonies were maintained under controlled conditions of 27 ± 2 °C, 80 ± 10% RH, and a 12-hour photoperiod. Upon pupation, replicates were transferred to Bugdorm cages (30 × 30 × 30 cm) for adult emergence. Emerged adults were fed on 10% sucrose solution, and females were blood-fed on day five using the wax pot-collagen membrane technique. Oviposition cups (200 mL) were provided three days post-blood feeding and retained for two days. Egg laid papers from the ovitraps were collected on the third day, air-dried at 27 ± 1 °C and 80 ± 10% RH, and stored in sealed containers with saturated potassium chloride solution to maintain humidity. The entire rearing procedure was replicated identically for the F1 generation.

Specifically, this study assessed wing length, body weight (measured individually) in the strains *w*Mel and *w*AlbB *Ae. aegypti* (Pud) in comparison with the uninfected *Ae. aegypti* (Pud) laboratory strain and the density of *Wolbachia*. An overview of the study design and methodology is presented as a flowchart in **Figure 1**.

### 2.3 Larval diets

Four commercially available larval diets were selected for this study considering local accessibility and cost-effectiveness in the Indian context: (1) fish feed (TetraBits, Tetra, Germany; LD1), (2) Laboratory Rodent Diet (VRK Nutritional Solutions, Pune, India; LD2), (3) Mushroom powder derived from 100% natural button mushroom, *Agaricus bisporus* (Transdelta Agro Products, Maharashtra, India; LD3), and (4) a 2:3 mix of Brewer’s yeast and Dog biscuit (LD4). Dog biscuits provide proteins, animal fat, calcium, and vitamins, while brewer’s yeast is rich in proteins, starch, sugars, and minerals. Mushroom powder offers a plant-based nutritional source containing proteins, carbohydrates, vitamins, minerals, dietary fibers, and various bioactive compounds, which may contribute to larval growth and development. Prior to feeding, LD1 granules and LD2 pellets were dried and ground into powder. Each diet was measured and provided ~0.06 g of food per larva (same quantity for all instars), with food provided on alternate days (Jeffrey Gutiérrez et al. (39). All experiments were initiated simultaneously, with three replicates (100 larvae/replicate) per mosquito strain per diet (4 diets × 3 strains × 3 replicates), and trays were monitored regularly for evaporation and larval feed intake. The tray size was 45 cm Length × 30 cm Width × 5 cm Height, with each tray carrying 3 liters of water. The experimental setup was maintained at 27 ± 2 °C temperature and 80 ± 10% Relative Humidity (RH) under a 12-hour photoperiod.

### 2.4 Fitness characteristics

#### 2.4.1 Wing length

Wing length serves as a proxy for mosquito fitness, as it is proportional to overall body size (40). To assess wing length, ten one-day-old unfed male and ten female mosquitoes, from the *w*Mel and *w*AlbB *Ae. aegypti* (Pud) strains, as well as the laboratory-reared uninfected *Ae. aegypti* (Pud) strains corresponding to each diet treatment were immobilized at minus 20 degree Celsius for 10 minutes. The right wing of all specimens was carefully detached and mounted on a glass slide. Wing length was measured from the alular notch (axillary incision) to the wing tip, excluding fringe scales, using a stereomicroscope (Olympus SZ61) fitted with a digital camera (Olympus DP22) and imaging software (cellSens Entry 1.13) by a single observer. Three independent biological replicates were performed for each diet-species combination.

#### 2.4.2 Body weight

For body weight estimation, ten one-day-old unfed females and unfed males from each *Wolbachia* transinfected *Ae. aegypti* (*w*Mel and *w*AlbB) and the uninfected *Ae. aegypti* (Pud) strains were collected from each replicate under each diet treatment. Mosquitoes were euthanized using ethanol exposure and individually transferred to pre-weighed 1.5 ml sterile microcentrifuge tubes (MCT). The samples were then dried at 60 °C using a dry bath for 12 hours, after which the dry body weight of each mosquito was measured individually using a semi-micro analytical balance with a readability of 0.01 mg (0.00001g).

The fitness characteristics such as body weight and wing length were measured individually for each mosquito.

#### 2.4.3 *Wolbachia* density

To investigate variability in maternal transmission of *Wolbachia* among progeny of transinfected *Ae. aegypti* strains (*w*Mel and *w*AlbB) reared under different dietary conditions, *Wolbachia* density and transmission frequency were evaluated through molecular screening. These endosymbionts spread through maternal inheritance, i.e., infected females pass them to their progeny, making transmission fidelity a key factor in the success of population replacement efforts DNA was isolated from all mosquito strains for the molecular screening of *Wolbachia*. Each mosquito was homogenized in 50 µL of DNA extraction buffer containing squash buffer (10 mM Tris base, 1 mM EDTA, 25 mM NaCl, pH 8.2) and proteinase K (15 mg/ml) in a 1:80 ratio using a tissue lyser (Qiagen, Germany). The homogenate was incubated for 5 min at 56 °C, followed by incubation at 98 °C for 15 min to inactivate the enzyme. The resulting extract was then used as the template for duplex real-time PCR targeting either *Wolbachia* surface protein (wsp) gene specific to *w*Mel or *Wolbachia* ankyrin repeat domain specific to *w*AlbB and the housekeeping gene ribosomal protein 17 (RPS17) specific to *Ae. aegypti*.

The primers used for *w*Mel (*wspTM2*); the forward primer was 5′-CATTGGTGTTGGTGTTGGTG-3′, the reverse primer was 5′-ACACCAGCTTTTACTTGACCAG-3′, and the probe was LC640-TCCTTTGGAACCCGCTGTGAATGA-LowaBLACK. For *w*AlbB (*Alb_16009*), the forward primer was 5′-AGTAGTGCAGCGAGTCT-3′, the reverse primer was 5′-TGGAGGAAGAGTTCACTGTGC-3′, and the probe was FAM-ZEN-AATTATCCCCTACCAAAGCAATTAAGATAGAAT-LowaBLACK. For the reference gene *Rps17* (*Ae. aegypti*), the forward primer was 5′-TCCGTGGTATCTCCATCAAGCT-3′, the reverse primer was 5′-CACTTCCGGCACGTAGTTGTC-3′, and the probe was HEX-CAGGAGGAGGAACGTGAGCGCAG-BHQ1 (31). All assays were run under the following thermal cycling conditions: pre-incubation at 95 °C for 5 min (1 cycle); amplification for 45 cycles of 95 °C for 10 s, 60 °C for 15 s, and 72 °C for 1s; followed by cooling at 40 °C for 10 s (1 cycle). The reaction volume was 10 µL, containing 1× LightCycler 480 Probe Master (Roche, Switzerland), primers and probes for the target and housekeeping genes at final concentrations of 0.25 µM each, 1µL of DNA template and 2.80µL of nuclease-free water (**Supplementary Table 3**). The relative density of *Wolbachia* was determined for both *w*Mel and *w*AlbB by delta Ct method (2^-ΔCt^) using RPS17 as the reference gene.

#### 2.4.4 Randomization, measurement procedure and quality control

To minimize confounding due to batch, positional, and handling effects, randomization was applied at multiple stages of the experiment. The first-instar larvae used to establish each replicate were randomly selected from pooled egg batches of the same parental cohort for each strain, prior to allocation into diet treatment trays. In addition, rearing trays for all diet–strain–generation combinations were randomly assigned to positions within the insectary shelving and rotated at regular intervals (e.g., every 2–3 days) to control for potential microclimate variation (temperature/humidity gradients, light exposure) across the rearing room. All morphometric and molecular measurements were performed as per the standardized GLP protocols. Adults selected for wing length, body weight, and *Wolbachia* density measurement were randomly sampled from each replicate cage. The calibration of the equipment such as electronic weighing balance for body weight and thermal cycler for qPCR reaction was ensured prior to the study. Insectary temperature and humidity were continuously logged throughout the rearing period to confirm adherence to the target conditions (27 ± 2 °C, 80 ± 10% RH).

### 2.5 Statistical analysis

Continuous outcome variables (mosquito fitness characteristics and *Wolbachia* density) were summarized as mean ± standard deviation. The experimental unit for statistical analysis was the biological replicate corresponding to a specific diet–strain–generation combination. The effect of larval diet, mosquito strain, and generation on fitness parameters were assessed using Linear model. Male and female mosquito characteristics were modelled separately. No imputation procedures were applied, as the missingness involved an entire treatment cell rather than isolated observations. Model assumptions, including normality of residuals and homogeneity of variance, were assessed prior to analysis.

Separate linear models were fitted for each outcome (male wing length, female wing length, male body weight, and female body weight) to evaluate the effects of diet, strain, generation, and their interactions. Eight biologically plausible candidate models were specified *a priori* for each outcome, beginning with models containing individual main effects and progressively incorporating two-way and three-way interaction terms. Model performance was compared using the Akaike Information Criterion (AIC), which balances model fit against model complexity. The model with the lowest AIC was considered the most parsimonious and was selected as the preferred model for inference. Statistical significance of the retained fixed effects was assessed using analysis of variance, and two-sided p-values <0.05 were considered statistically significant. Various Model selection was based on Akaike Information Criterion (AIC), with preference given to the most parsimonious model providing the best fit.

*Post-hoc* pairwise comparisons were performed using Tukey’s adjustment method when significant effects were detected. All statistical analyses were conducted using R version 4.2.2 (41). Linear mixed-effects models were fitted using the lme4 package (42), and estimated marginal means were obtained using the *emmeans* package (43).

## 3 Results

### 3.1 Fitness characteristics

This laboratory experimental study comprised four larval diet treatments, two generations (F0, F1), three mosquito strains (Control, wAlbB, and wMel), and three independent biological replicates, resulting in a total of 72 expected experimental observations (4 diets × 3 strains × 2 generations × 3 replicates) with an aim to study the effect of Diet on the mosquito characteristics, while evaluating the potential modifying effects of Wolbachia strain, generation, and their interactions. However, observations corresponding to LD2–F1–wMel combination were unavailable due to insufficient numbers of F1-generation mosquitoes and error/invalidation during the experiment.

#### 3.1.1 Wing length

**Figure 2:**
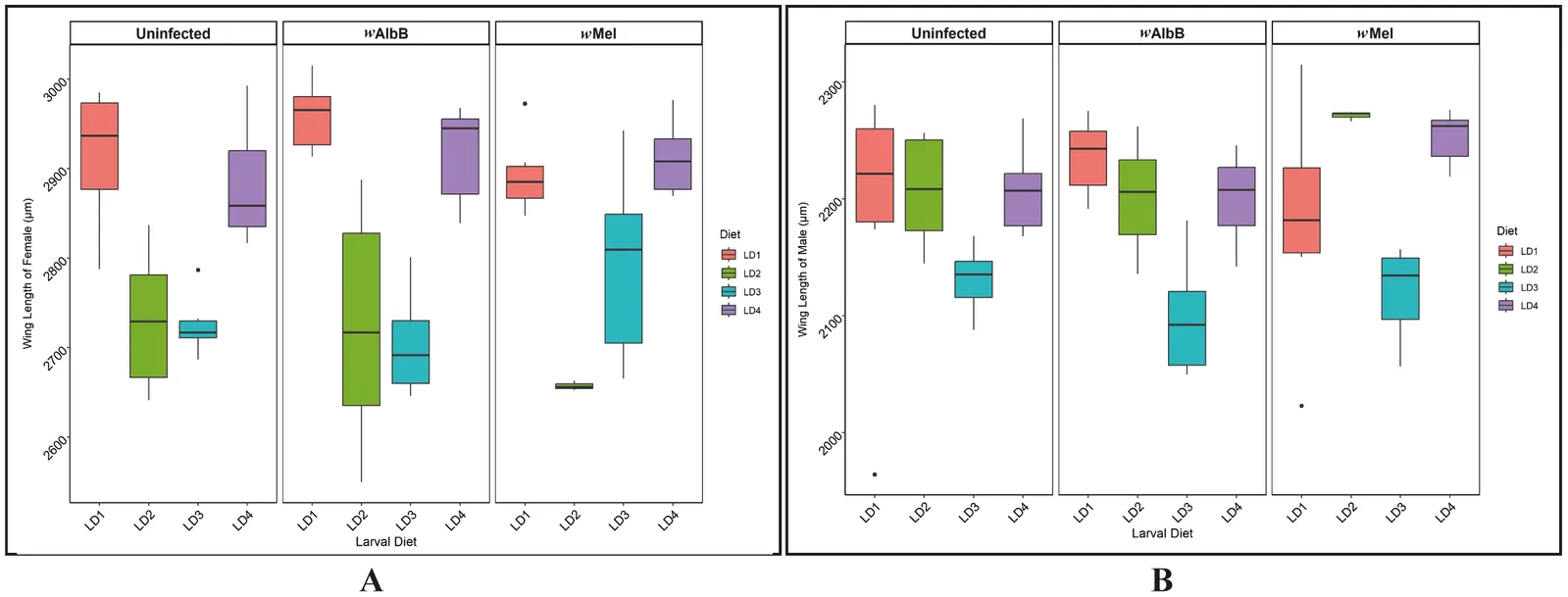
**(A)** Effect of diet on female wing length across various strains **(B)** Effect of larval diet on male wing length across *Aedes aegypti* strains.

Female wing length was consistently greater than male wing length across all diet groups and strains (**Table 1**). It varied across larval diets, generations, and *Wolbachia* strains (**Figure 2A**). Based on the estimated marginal means and the Tukey-adjusted pairwise comparisons: In the F0 generation, the LD2 diet yielded the highest mean wing length, although it did not differ significantly from LD1 or LD4; all three diets produced significantly larger wing lengths than LD3. In the F1 generation, the LD4 diet resulted in the highest mean wing length and was significantly superior to LD3, while differences from LD1 and LD2 were not statistically significant. The preferred model for female wing length revealed a significant three-way interaction among diet, strain, and generation (F = 3.98, P = 0.0044), indicating that the effect of larval diet differed according to both Wolbachia strain and generation. Significant Diet × Strain (P = 0.0135) and Diet × Generation (P < 0.001) interactions further supported this context-dependent response, whereas the Strain × Generation interaction was not significant (P = 0.1434) (**Table 2**). Therefore, diet-specific differences were examined within each strain–generation subgroup using Tukey-adjusted pairwise comparisons, presented in **Supplementary Table 1**.

**Table 1:** Distribution of mosquito fitness characteristics across various diets and *Aedes aegypti* strains.

Diet	Uninfected (n, 24)	*w*AlbB (n, 24)	*w*Mel (n, 21)
Wing length male in µm			
LD1	2187.68 ± 116.63	2235.95 ± 32.82	2181.11 ± 97.31
LD2	2207.18 ± 47.99	2201.49 ± 48.01	2271.12 ± 4.22
LD3	2130.95 ± 28.5	2098.69 ± 51.05	2120.08 ± 40.02
LD4	2207.31 ± 37.91	2200.48 ± 39.24	2252.66 ± 23.11
Wing length female in µm			
LD1	2914.66 ± 77.45	2959.6 ± 40.01	2893.15 ± 43.94
LD2	2730.04 ± 78.81	2723.88 ± 133.91	2657.03 ± 5.4
LD3	2725.03 ± 33.75	2703.78 ± 59.12	2793.14 ± 107.08
LD4	2882.33 ± 68.6	2917.06 ± 57.57	2911.59 ± 42.42
Body weight male, *in mg*			
LD1	2.527 ± 0.165	2.69 ± 0.341	2.468 ± 0.298
LD2	3.888 ± 0.366	2.742 ± 0.267	2.66 ± 0.036
LD3	3.055 ± 0.955	2.815 ± 0.301	2.31 ± 0.272
LD4	2.442 ± 0.161	2.893 ± 0.492	2.505 ± 0.104
Body weight female, *in mg*			
LD1	5.6 ± 1	6.22 ± 0.63	5.4 ± 0.66
LD2	6.4 ± 0.84	5.65 ± 1.02	5.2 ± 0.04
LD3	5.36 ± 0.98	5.52 ± 0.5	5.2 ± 0.56
LD4	5.66 ± 0.36	6.47 ± 0.99	5.92 ± 0.37

**Table 2:** Effects of larval diet and mosquito strain on mosquito fitness characteristics.

Mosquito fitness characteristics	Source	F statistic value	P value
Wing Length Male	Diet	14.11	<0.001
	Generation	0.12	0.7260
	Diet*Generation	5.58	0.0019
Wing Length Female	Diet	82.75	<0.001
	Strain	1.33	0.2731
	generation	50.62	<0.001
	Diet *Strain	3.05	0.0135
	Strain* Generation	2.03	0.1434
	Generation* Diet	7.12	<0.001
	Generation* Diet*strain	3.98	0.0044
Body Weight Male	Diet	16.15	<0.001
	Strain	20.43	<0.001
	generation	9.89	0.0029
	Diet *Strain	14.5	<0.001
	Strain* Generation	16.25	0.0036
	Generation* Diet	5.18	<0.001
	Generation* Diet*strain	8.95	<0.001
Body Weight Female	Diet	6.31	<0.001
	Strain	2.06	0.1388
	generation	1.58	0.2145
	Diet *Strain	3.43	0.0069
	Strain* Generation	7.79	0.0012
	Generation* Diet	19.84	<0.001
	Generation* Diet*strain	2.15	0.0767

**Figure 3:**
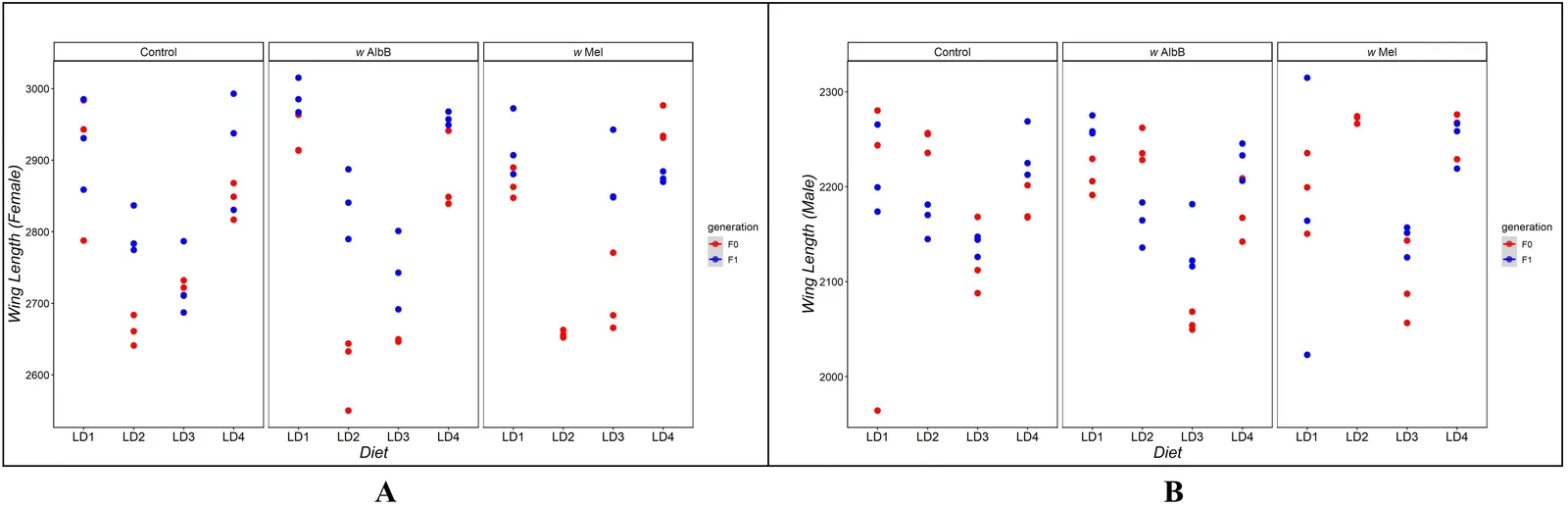
**(A)** Effect of diet on female wing length across various strains and generation **(B)** Effect of larval diet on male wing length across *Aedes aegypti* strains and generations.

In F0 generation, mosquitoes reared under LD2 and LD4 exhibited larger wing lengths compared to those reared under and LD3. Across most dietary treatments, F1 mosquitoes tended to show slightly greater wing lengths than F0 mosquitoes (**Figures 3A, B**). Similar dietary trends were observed across Uninfected, *w*AlbB, and *w*Mel groups, although the magnitude of differences varied between strains.

Male wing length ranged between 2099–2271 µm. LD3 consistently produced the shorter wing lengths across all strains (Uninfected: 2130.95, *w*AlbB: 2098.69, *w*Mel: 2120.08). It varied across diets, generation and mosquito strains, with relatively higher values observed under LD1 and LD2 compared to LD3 (**Figure 2B**). The preferred model for female wing length included diet, generation, and their interaction. Diet (F = 55.91, *P* < 0.001) and generation (F = 37.37, *P* < 0.001) significantly influenced female wing length. A significant Diet × Generation interaction (F = 5.32, *P* = 0.0025) indicated that the effect of larval diet differed between the F0 and F1 generations.

LD1 yields the longest female wings comparatively in wAlbB strain (2959.6 ± 40.01), while LD2 and LD3 tend to produce shorter wings. The wMel strain on LD2 diet showed the lowest female wing length (2657.03 ± 5.4) with an extremely low SD. Variability (SD) is highest in LD2 for Uninfected females (± 78.81) and in wAlbB on LD2 (± 133.91).

#### 3.1.2 Body weight

**Figure 4:**
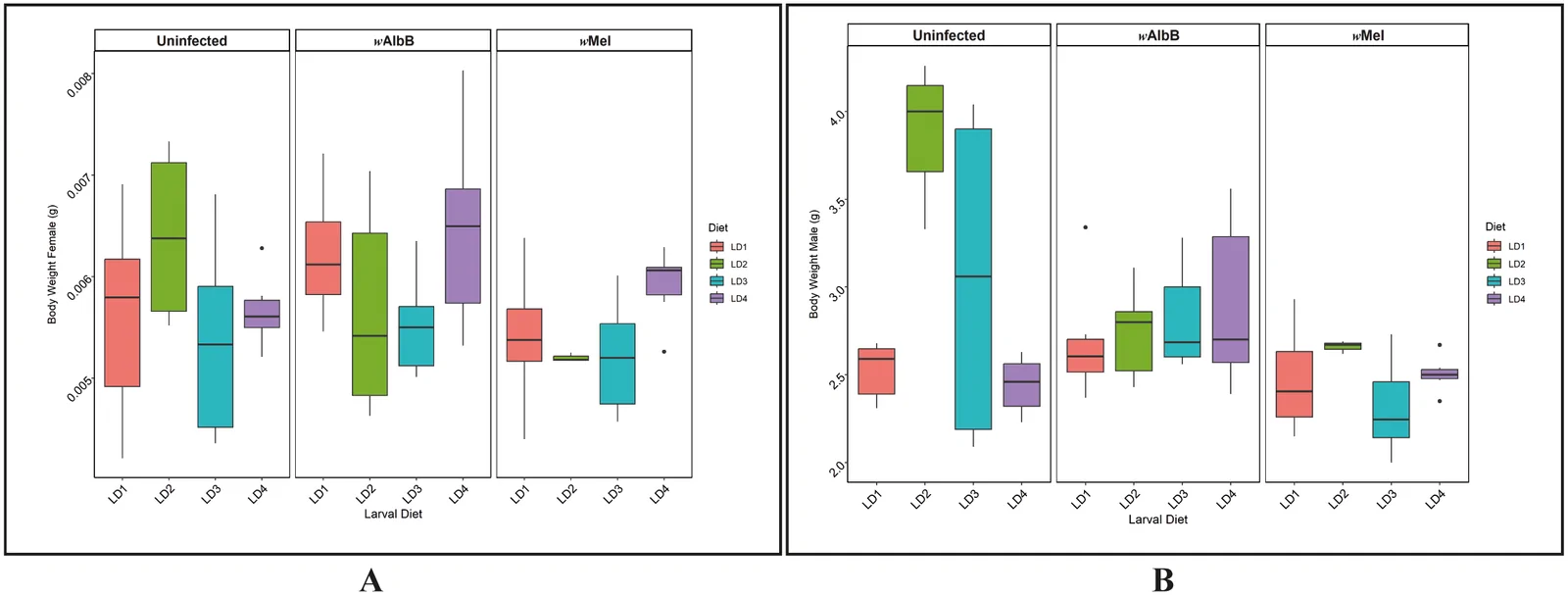
**(A)** Effect of diet on female body weight across various strains **(B)** Effect of larval diet on male body weight across *Ae. aegypti* strains.

Male body weight varied across both diets and strains. The Uninfected strain on LD2 has a high mean weight (3.888 ± 0.366mg), but the *w*AlbB and *w*Mel strains on the same diet were considerably lighter (2.742 and 2.660 mg, respectively) (**Figure 4A**). significant Diet × Strain (*F* = 14.50, *P* < 0.001), Strain × Generation (*F* = 16.25, *P* = 0.0036), and Diet × Generation (*F* = 5.18, *P* < 0.001) interactions indicated that the influence of larval diet on male body weight varied across both Wolbachia strains and generations. Consequently, the effects of diet were examined separately within each strain–generation combination using Tukey-adjusted pairwise comparisons (**Supplementary Table 1**).

*w*AlbB females on LD1 (6.22 ± 0.63) and LD4 (6.47 ± 0.99) were found to have higher body weight, with LD4 being the highest value in the entire female dataset. Female body weight was best explained by the three-way interaction model. Although the Diet × Strain × Generation interaction was not statistically significant (*F* = 2.15, *P* = 0.0767), significant Diet × Strain (*P* = 0.0069), Diet × Generation (*P* < 0.001), and Strain × Generation (*P* = 0.0012) interactions indicated that the effect of larval diet varied independently with Wolbachia strain and generation. Therefore, dietary effects were examined within the relevant strain and generation combinations using Tukey-adjusted pairwise comparisons (**Supplementary Table 1**).

**Figure 5:**
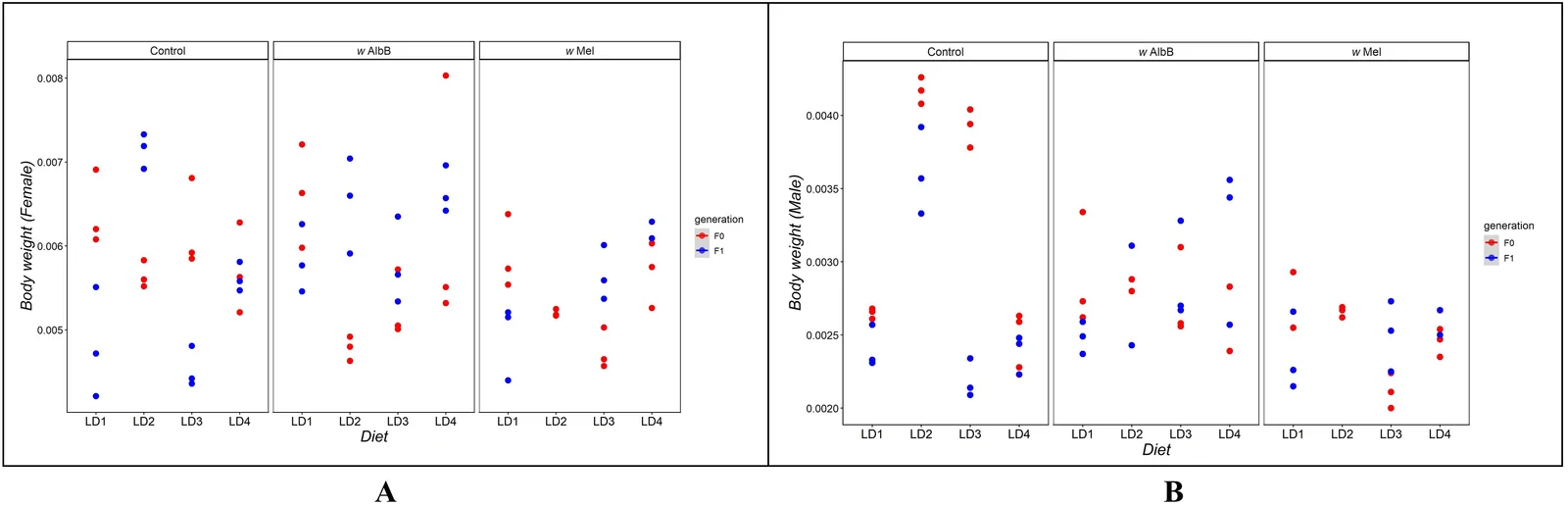
**(A)** Effect of diet on female body weight across various strains and generation **(B)** Effect of larval diet on male body weight across *Aedes aegypti* strains and generations.

Female body weight showed comparatively smaller variation across diets and strains. Neither diet nor mosquito strain had a statistically significant effect on female body weight (p > 0.05) (**Figure 4B**). Generation-level variance was negligible, and therefore a simple linear model was fitted for this outcome (**Figures 5A, B**).

Overall, larval diet played a major role in determining mosquito fitness characteristics, particularly wing length and male body weight. While wing length was primarily influenced by diet, male body weight was affected by both diet and mosquito strain, with evidence of strain-dependent dietary responses.

#### 3.1.3 *Wolbachia* density

**Figure 6:**
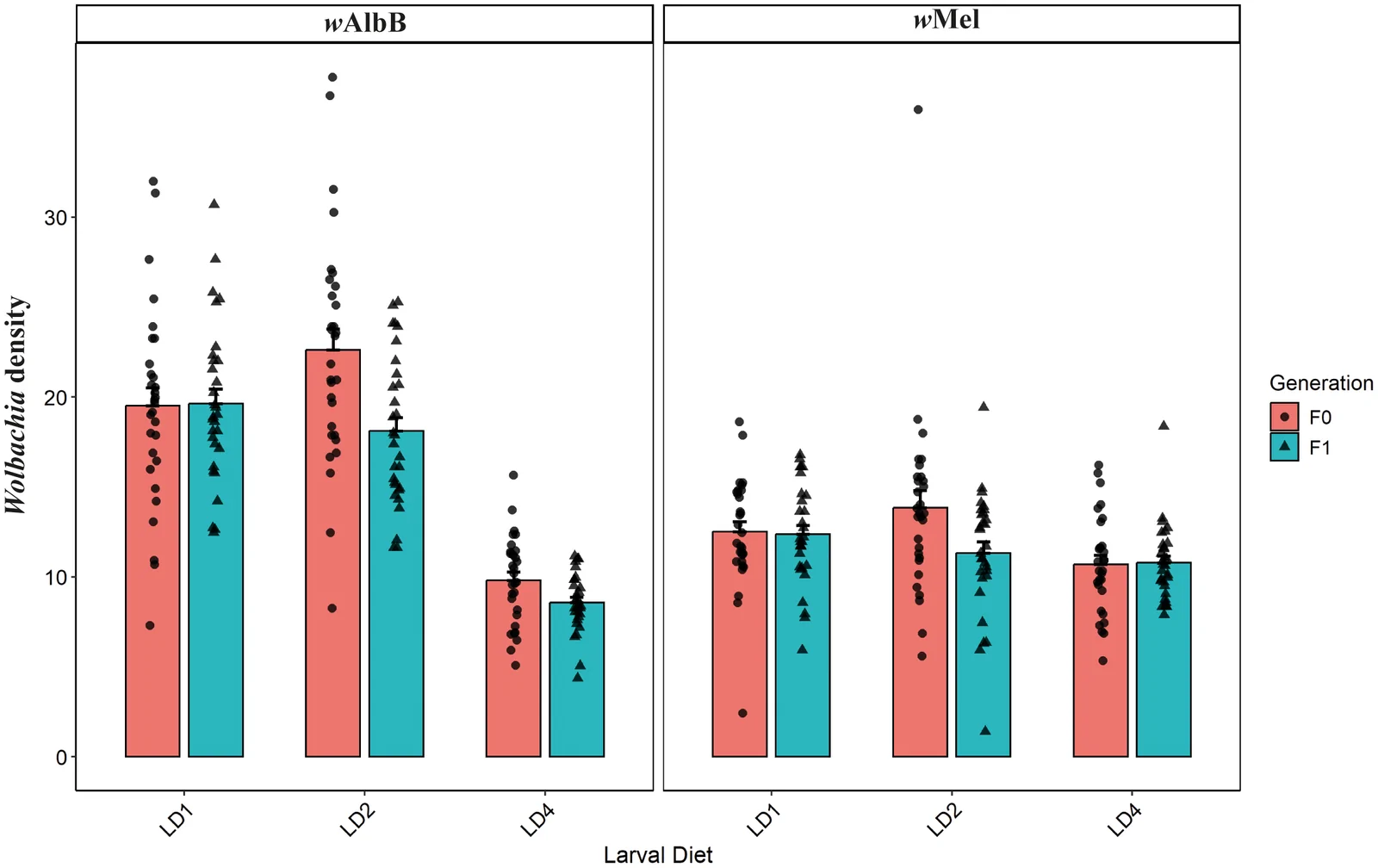
Comparison of *Wolbachia* density across three diets, strains and generations.

Descriptive analysis showed substantial variation in *Wolbachia* density across larval diets, generations, and mosquito strains (**Table 3**). Overall, the *w*AlbB strain exhibited higher *Wolbachia* densities than the *w*Mel strain across most diet treatments. Higher densities were generally observed in mosquitoes reared on fish feed (LD1) and laboratory rodent diet (LD2), whereas mosquitoes reared on the dog biscuit and brewer’s yeast diet (LD4) exhibited comparatively lower *Wolbachia* densities (**Figure 6**).

**Table 3:** Distribution of *Wolbachia* density across various generations, diets, and *Aedes aegypti* strains.

Diet	Generation	Statistics	*w*AlbB	*w*Mel
Fish Feed (LD1)	F0	Mean ± SD	19.51 ± 5.52	12.51 ± 3.05
		Min	7.31	2.45
		Max	32.00	18.64
	F1	Mean ± SD	19.65 ± 4.42	12.37 ± 2.79
		Min	12.47	5.94
		Max	30.70	16.80
Laboratory Rodent Diet (LD2)	F0	Mean ± SD	22.62 ± 6.41	13.85 ± 5.24
		Min	8.28	5.62
		Max	37.79	36.00
	F1	Mean ± SD	18.11 ± 4.13	11.32 ± 3.43
		Min	11.63	1.40
		Max	25.28	19.43
Dog Biscuit with Brewer’s Yeast (LD4)	F0	Mean ± SD	9.82 ± 2.47	10.71 ± 2.72
		Min	5.10	5.35
		Max	15.67	16.22
	F1	Mean ± SD	8.58 ± 1.64	10.79 ± 2.09
		Min	4.38	7.89
		Max	11.16	18.38

A linear mixed-effects model was fitted to assess the effect of larval diet and *Aedes aegypti* strain on *Wolbachia* density, with generation included as a random effect. As the mushroom powder treatment did not show any detectable *Wolbachia* density in any of the replicates, it was excluded from the analysis. The reference category was the *w*AlbB strain reared under the LD1 diet. The estimated mean *Wolbachia* density for the reference group was 19.58 ± 0.83. Compared to LD1, the LD2 diet showed no significant change in density among the *w*AlbB strain (β = 0.79, *p* = 0.280), whereas the LD4 diet showed a significant reduction in density (β = −10.38, *p* < 0.001).

The *w*Mel strain exhibited significantly lower *Wolbachia* density than *w*AlbB under the LD1 diet (β = −7.14, *p* < 0.001). The interaction between LD2 and *w*Mel was not statistically significant (β = −0.64, *p* = 0.532), indicating that the effect of LD2 did not differ between strains. However, the interaction between LD4 and *w*Mel was significant and positive (β = 8.69, *p* < 0.001), suggesting that the reduction in *Wolbachia* density observed under LD4 was less pronounced in the *w*Mel strain compared to *w*AlbB.

## 4 Discussion

The role of larval diet is an important factor to determine as it directly impacts the larval and adult development and fitness. During the aquatic developmental phase, mosquitoes acquire essential nutrients that provide the foundation for successful metamorphosis and adult emergence. This nutritional accumulation supports pupal transformation and adult formation, ultimately affecting critical traits including lifespan, reproductive capacity, and pathogen transmission potential (44–46). The quality and quantity of larval nutrition influence not only life table parameters but also establish the metabolic framework underlying adult survival strategies and reproductive success (47, 48). Our previous investigation (32) demonstrated the effects of four distinct larval diets on life table traits, reproductive capacity, and survival probability across different strains of *Ae. aegypti*. This study revealed that LD1 and LD4 diets significantly enhanced these morphological traits compared to other nutritional regimens. Furthermore, the interaction between larval nutrition and bacterial endosymbionts such as *Wolbachia* adds additional complexity to our understanding of how dietary factors influence mosquito development.

The current study assessed morphological characteristics among *Wolbachia*-transinfected *Ae. aegypti* strains (*w*Mel and *w*AlbB) and uninfected control populations maintained under laboratory conditions. Results indicate that larval nutritional regimens produce sex-specific and strain-dependent effects on key parameters, including wing length, body weight, and bacterial density, across both uninfected and *Wolbachia*-transinfected mosquito strains.

The significant interaction between larval diet and mosquito strain for male body weight suggests that the effect of diet is strain-dependent. This finding indicates that different *Wolbachia*-infected strains may vary in their ability to utilize or allocate nutrients acquired during larval development. Such strain-specific responses could arise from differences in metabolic demands associated with *Wolbachia* infection, host-symbiont interactions, or variation in nutrient assimilation efficiency among strains. From an operational perspective, these results suggest that a single larval diet may not be equally optimal for all strains and that diet formulations may need to be tailored to individual strains to maximize male quality for *Wolbachia*-based vector control programs. The importance of this research lies in optimizing protocols for rearing healthy *Wolbachia*-transinfected *Ae. aegypti* strains for mass production programs. Maintaining robust laboratory-reared mosquitoes is essential for their successful competition with wild populations and effective dissemination of *Wolbachia* in field environments.

### 4.1 Impact of diet on morphological traits

In the current study, the larval diet played a major role in wing length and male body weight. While wing length was primarily influenced by diet, male body weight was affected by both diet and mosquito strain, with evidence of strain-dependent dietary responses, which is consistent with previous studies (49, 50). Mosquito body size is considered a significant life history trait due to its strong association with various fitness-related characteristics, as well as its influence on susceptibility to infection and disease dissemination (12).

In males, diet significantly affected wing length across different diet-generation combination, F 5.58, 0.0019). *Wolbachia* strains had no significant independent effect in either sex, and F1 females tended to show slightly larger wings than F0. The consistent pattern of larger female wing measurements compared to males across all feeding regimens matches documented sexual size differences in prior research (44, 45). Hence, the larval diet quality was the key determinant of wing size, with poorer diets consistently associated with reduced wing length. These findings highlight the dominant role of larval nutrition in shaping mosquito body size, as no significant difference in wing length was observed between uninfected and *Wolbachia*-transinfected *Ae. aegypti*, suggesting that *Wolbachia* infection does not alter the nutritional influence on this trait.

Beyond wing length, male body weight was significantly influenced by both larval diet and mosquito strain, with a significant diet-strain interaction indicating strain-dependent dietary responses. Importantly, LD2 produced higher body weight than LD1 across several strain comparisons. In contrast, female body weight was unaffected by either diet or strain (p > 0.05) independently. For wing length and body weight, both LD1 and LD4 consistently supported larger wing lengths in females, suggesting better nutritional support for body size development. Overall, larval diet was a key determinant of mosquito morphological traits, particularly wing length and male body weight, with the latter further modulated by mosquito strain (51, 52). This pattern is consistent with reports that *w*Mel and *w*AlbB differentially affect *Ae. aegypti* fitness traits, with *w*Mel-transinfected mosquitoes generally exhibiting greater fitness costs (reduced fecundity, blood feeding, and *Wolbachia* density) than *w*AlbB-infected mosquitoes (53). Similarly, larval diet manipulation has been shown to mitigate *Wolbachia*-associated fitness costs more effectively in *w*AlbB-infected lines than in *w*MelM-infected lines, where reduced fecundity persisted despite adequate wing length, a pattern attributed to *w*Mel for greater reliance on host-derived lipids and amino acids. This supports our interpretation that the observed diet and strain interaction on body weight reflects genuine, strain-specific differences in nutritional demand rather than a generalized dietary effect.

Across most larval diets, F1 mosquitoes tended to have slightly greater wing lengths than F0 mosquitoes for wing length. Similarly, for body weight traits, the generation-level variance was negligible. The absence of significant differences in the morphological traits among the dietary groups suggests that larval diet did not substantially influence mosquito morphological traits across the F0 and F1 generations. These findings indicate that generational variation in the assessed morphological traits was minimal, regardless of the diet examined.

We detected no adult survival in the *w*Mel *Ae. aegypti* strain in all the three replicates, and were therefore unable to assess wing length or body weight for this combination under LD2 diet. Similar findings have been reported previously, where a nutritionally imbalanced or single-source larval diet resulted in complete failure to pupate, suggesting toxicity or critical nutrient deficiency (50). Given that larval nutrition is a well-established determinant of adult body size and fitness traits, including wing length (46), the loss of this treatment combination limits our ability to assess diet-mediated variation in these parameters for the F1 generation. Furthermore, host nutritional status has been shown to modulate *Wolbachia*-associated phenotypes, including pathogen interference and physiological stress responses (54), and diet formulation is increasingly recognized as an important factor shaping *Wolbachia* density and efficacy in mosquito-based control strategies (55). Therefore, the missing LD2-F1-wMel combination may unclear diet-generation-specific effects on *Wolbachia* density that warrant further investigation.

In addition to wing length and body weight, the *Wolbachia* density across different diets revealed that both larval diet and mosquito strain significantly influenced *Wolbachia* density. Within the *w*AlbB strain, LD2 produced no significant change in density compared to LD1, while LD4 led to a significant reduction. The *w*Mel strain generally exhibited lower *Wolbachia* density than *w*AlbB, and although LD4 also reduced density in *w*Mel, this reduction was less prominent compared to *w*AlbB. Overall, these findings suggest that larval diet, particularly LD4, differentially affects *Wolbachia* density in a strain-dependent manner. Studies have shown that maintaining stable *Wolbachia* infections is essential for the successful application of *Wolbachia*-based biocontrol strategies against dengue and malaria vectors (56, 57). Variations in *Wolbachia* density can influence key phenotypic traits, including cytoplasmic incompatibility, pathogen blocking, and maternal transmission, thereby affecting the effectiveness and sustainability of *Wolbachia* dissemination in target mosquito populations (58, 59).

### 4.2 Implication on *Wolbachia*-based vector control strategies

The study findings reveal that the *Wolbachia* transinfected and uninfected *Ae. aegypti* varies in their nutritional requirements or metabolic efficiency in converting dietary resources into somatic growth. This is consistent with evidence that *Wolbachia* directly competes with its mosquito host for amino acids (60), and that the lack of independent fatty acid and cholesterol synthesis pathways of *Wolbachia* drives further reliance on host-derived lipid resources (61). Such resource competition could impose an additional metabolic burden on *Wolbachia*-infected strains, mainly under suboptimal dietary conditions, potentially explaining the diet-dependent divergence in body weight observed between strains in this study. This is further supported by evidence that host nutritional status can modulate *Wolbachia*-associated physiological phenotypes more broadly (54).

From a practical viewpoint, this finding has direct implications for *Wolbachia*-based mass-rearing and release programmes, where standardized larval diets are often applied uniformly across strains. Our results suggest that diet formulations may need to be strain-specific rather than generalized, to ensure optimal larval development, adult body size, and *Wolbachia* density are simultaneously achieved. Future studies optimizing diet formulations for individual *Wolbachia* strains may improve the efficiency and consistency of mass-rearing pipelines.

These findings are consistent with our earlier work (Yazhini et al., 2025), and together both studies support the adoption of LD1 and LD4 as, practical diets for mass-rearing facilities in India. Across life table traits, fecundity, survival, mosquito fitness, and *Wolbachia* density, LD1 and LD4 diets demonstrated superior performance for both *Wolbachia*-free and transinfected strains of *Ae. aegypti* under laboratory conditions.

### 4.3 Limitations of the study

As noted in Yazhini et al. (32), one limitation of this study is the absence of a formal sample size calculation and power analysis, as the major part of this work was conducted simultaneously. The primary objective was to assess the feasibility and biological plausibility of the diets under evaluation, and in accordance with WHO guidelines for bioassay procedures, 100 third-instar larvae per replicate were used across each of the three *Wolbachia* strains, providing a standardized and methodologically justified basis for comparison. Beyond this, the study was conducted under controlled laboratory conditions and has not been field-validated, so responses may differ under natural environmental variability. The nutritional composition of the four diets was likewise not analysed, limiting the attribution of observed effects to specific dietary components, and only two generations (F0 and F1) were examined, which constrains assessment of longer-term effects. Finally, the LD2-F1-*w*Mel combination was excluded owing to high adult mortality; although the statistical model accommodates unbalanced designs, this reduces confidence in the corresponding interaction estimates and may itself indicate that this diet is nutritionally inadequate for *w*Mel-infected *Ae. aegypti*.

## 5 Conclusion

This study validated larval nutrition significantly influences morphological traits and *Wolbachia* density of *Ae. aegypti* mosquitoes across both *Wolbachia*-transinfected and uninfected strains, with diets LD1 and LD4 consistently supporting optimal wing development and overall traits compared to LD2 and LD3. While male body weight showed complex strain-dependent dietary responses, female body weight remained stable across treatments, indicating sex-specific nutritional sensitivities. The LD4 diet resulted in reduced *Wolbachia* density through unknown mechanisms and, LD1 diet produced superior outcomes, making them the recommended larval diet for mass-rearing programs intended for vector control field releases. These findings highlight the importance of standardized nutritional protocols for maintaining healthy, competitive mosquito colonies and suggest future research priorities, including the development of cost-effective, locally-sourced diet formulations for Indian settings and investigations into the complex interactions between dietary components, native microbiota, and *Wolbachia* density patterns to optimize both mosquito fitness and symbiont stability for successful population replacement and suppression strategies.

## Data Availability

The original contributions presented in the study are included in the article/**Supplementary Material**. Further inquiries can be directed to the corresponding author.

## Supplementary material

Supplementary Table 1 Tukey’s *Post-hoc* pairwise comparisons of diet effects on mosquito lines across strains.

Supplementary Table 2 Linear mixed effects model summary of various fitness characteristics.

Supplementary Table 3 Details of final primer and probe concentrations used in each reaction for detecting *Wolbachia* density.
