# Supplementary material for: Larval diet-mediated morphological trait and Wolbachia density variation in Wolbachia-transinfected (wMel and wAlbB) and Wolbachia-free Aedes aegypti mosquitoes
Source: Front Insect Sci. 2026 Sep 3;6:1887426. doi: 10.3389/finsc.2026.1887426 (PMC13582537; doi:10.3389/finsc.2026.1887426)
Supplement: Supplementary file 1 [file Table1.docx]

**Supplementary Table 1: Tukey’s Post-hoc pairwise comparisons of diet effects on mosquito lines across generations and strains**

| **Subgroups** | **Diet Combination** | **Contrast (95% CI)** | **Statistical Significance** |
| --- | --- | --- | --- |
| **Wing Length Male** | | | |
| F0 | LD1 - LD2 | -65.24 (-143.84, 13.36) | NS |
|  | LD1 - LD3 | 96.85 (18.25, 175.45) | 0.006^*^ |
|  | LD1 - LD4 | -14.38 (-92.98, 64.21) | 0. NS |
|  | LD2 - LD3 | 162.09 (83.49, 240.69) | <0.001^*^ |
|  | LD2 - LD4 | 50.86 (-27.74, 129.45) | 0. NS |
|  | LD3 - LD4 | -111.24 (-189.83, -32.64) | 0.001^*^ |
| F1 | LD1 - LD2 | 51.16 (-36.72, 139.03) | 0. NS |
|  | LD1 - LD3 | 73.16 (-5.44, 151.76) | NS |
|  | LD1 - LD4 | -22.76 (-101.36, 55.84) | NS |
|  | LD2 - LD3 | 22.00 (-65.87, 109.88) | NS |
|  | LD2 - LD4 | -73.92 (-161.79, 13.96) | NS |
|  | LD3 - LD4 | -95.92 (-174.52, -17.32) | 0.007^*^ |
| **Wing Length Female** | | | |
| Strain = Uninfected &  generation = F0 | LD1 - LD2 | 242.8 (179.3,306.2) | <.0001 |
|  | LD1 - LD3 | 182.7 (119.3,246.2) | <.0001 |
|  | LD1 - LD4 | 60.1 (-3.4,123.5) | NS |
|  | LD2 - LD3 | -60.1 (-123.5,3.4) | NS |
|  | LD2 - LD4 | -182.7 (-246.1,-119.2) | <.0001 |
|  | LD3 - LD4 | -122.6 (-186.1,-59.2) | 0.015 |
| Strain = wAlbB, generation = F0 | LD1 - LD2 | 321.6 (258.1,385) | <.0001 |
|  | LD1 - LD3 | 267.7 (204.3,331.2) | <.0001 |
|  | LD1 - LD4 | 54.1 (-9.4,117.6) | NS |
|  | LD2 - LD3 | -53.8 (-117.3,9.6) | NS |
|  | LD2 - LD4 | -267.5 (-330.9,-204) | <.0001 |
|  | LD3 - LD4 | -213.6 (-277.1,-150.2) | <.0001 |
| Strain = wMel, generation = F0 | LD1 - LD2 | 209.5 (146,272.9) | <.0001 |
|  | LD1 - LD3 | 160.1 (96.6,223.5) | 0.0006 |
|  | LD1 - LD4 | -80.7 (-144.1,-17.2) | NS |
|  | LD2 - LD3 | -49.4 (-112.9,14) | NS |
|  | LD2 - LD4 | -290.2 (-353.6,-226.7) | <.0001 |
|  | LD3 - LD4 | -240.7 (-304.2,-177.3) | <.0001 |
| Strain = Uninfected, generation = F1 | LD1 - LD2 | 126.5 (63,189.9) | 0.0086 |
|  | LD1 - LD3 | 196.6 (133.1,260) | <.0001 |
|  | LD1 - LD4 | 4.6 (-58.9,68) | NS |
|  | LD2 - LD3 | 70.1 (6.6,133.5) | NS |
|  | LD2 - LD4 | -121.9 (-185.4,-58.5) | 0.0121 |
|  | LD3 - LD4 | -192 (-255.4,-128.5) | <.0001 |
| Strain = wAlbB, generation = F1 | LD1 - LD2 | 149.9 (86.4,213.3) | 0.0014 |
|  | LD1 - LD3 | 243.9 (180.5,307.4) | <.0001 |
|  | LD1 - LD4 | 31 (-32.5,94.4) | NS |
|  | LD2 - LD3 | 94 (30.6,157.5) | 0.0757 |
|  | LD2 - LD4 | -118.9 (-182.3,-55.4) | 0.014 |
|  | LD3 - LD4 | -212.9 (-276.4,-149.5) | <.0001 |
| Strain = wMel, generation = F1 | LD1 - LD3 | 40 (-23.5,103.4) | NS |
|  | LD1 - LD4 | 43.8 (-19.7,107.3) | NS |
|  | LD3 - LD4 | 3.8 (-59.6,67.3) | NS |
| **Body Weight Male** | | | |
| Strain = Uninfected &  generation = F0 | LD1 - LD2 | -1.52 (-1.853,-1.187) | <.0001 |
|  | LD1 - LD3 | -1.27 (-1.603,-0.937) | <.0001 |
|  | LD1 - LD4 | 0.15 (-0.183,0.483) | NS |
|  | LD2 - LD3 | 0.25 (-0.083,0.583) | NS |
|  | LD2 - LD4 | 1.67 (1.337,2.003) | <.0001 |
|  | LD3 - LD4 | 1.42 (1.087,1.753) | <.0001 |
| Strain = wAlbB, generation = F0 | LD1 - LD2 | 0.07 (-0.263,0.403) | NS |
|  | LD1 - LD3 | 0.15 (-0.183,0.483) | NS |
|  | LD1 - LD4 | 0.3 (-0.033,0.633) | NS |
|  | LD2 - LD3 | 0.08 (-0.253,0.413) | NS |
|  | LD2 - LD4 | 0.23 (-0.103,0.563) | NS |
|  | LD3 - LD4 | 0.15 (-0.183,0.483) | NS |
| Strain = wMel, generation = F0 | LD1 - LD2 | -0.08 (-0.413,0.253) | NS |
|  | LD1 - LD3 | 0.463 (0.13,0.796) | NS |
|  | LD1 - LD4 | 0.127 (-0.206,0.46) | NS |
|  | LD2 - LD3 | 0.543 (0.21,0.876) | 0.0419 |
|  | LD2 - LD4 | 0.207 (-0.126,0.54) | NS |
|  | LD3 - LD4 | -0.337 (-0.67,-0.004) | NS |
| Strain = Uninfected, generation = F1 | LD1 - LD2 | -1.203 (-1.536,-0.87) | <.0001 |
|  | LD1 - LD3 | 0.213 (-0.12,0.546) | NS |
|  | LD1 - LD4 | 0.02 (-0.313,0.353) | NS |
|  | LD2 - LD3 | 1.417 (1.084,1.75) | <.0001 |
|  | LD2 - LD4 | 1.223 (0.89,1.556) | <.0001 |
|  | LD3 - LD4 | -0.193 (-0.526,0.14) | NS |
| Strain = wAlbB, generation = F1 | LD1 - LD2 | -0.173 (-0.506,0.16) | NS |
|  | LD1 - LD3 | -0.4 (-0.733,-0.067) | NS |
|  | LD1 - LD4 | -0.707 (-1.04,-0.374) | 0.0047 |
|  | LD2 - LD3 | -0.227 (-0.56,0.106) | NS |
|  | LD2 - LD4 | -0.533 (-0.866,-0.2) | 0.0473 |
|  | LD3 - LD4 | -0.307 (-0.64,0.026) | NS |
| Strain = wMel, generation = F1 | LD1 - LD3 | -0.147 (-0.48,0.186) | NS |
|  | LD1 - LD4 | -0.2 (-0.533,0.133) | NS |
|  | LD3 - LD4 | -0.053 (-0.386,0.28) | NS |
| **Body Weight Female** | | | |
| Strain = Uninfected &  generation = F0 | LD1 - LD2 | 0.747 (0.06,1.434) | NS |
|  | LD1 - LD3 | 0.203 (-0.484,0.89) | NS |
|  | LD1 - LD4 | 0.69 (0.003,1.377) | NS |
|  | LD2 - LD3 | -0.543 (-1.23,0.144) | NS |
|  | LD2 - LD4 | -0.057 (-0.744,0.63) | NS |
|  | LD3 - LD4 | 0.487 (-0.2,1.174) | NS |
| Strain = wAlbB, generation = F0 | LD1 - LD2 | 1.823 (1.136,2.51) | 0.0003 |
|  | LD1 - LD3 | 1.347 (0.66,2.034) | 0.01 |
|  | LD1 - LD4 | 0.32 (-0.367,1.007) | NS |
|  | LD2 - LD3 | -0.477 (-1.164,0.21) | NS |
|  | LD2 - LD4 | -1.503 (-2.19,-0.816) | 0.0034 |
|  | LD3 - LD4 | -1.027 (-1.714,-0.34) | 0.0717 |
| Strain = wMel, generation = F0 | LD1 - LD2 | 0.683 (-0.004,1.37) | NS |
|  | LD1 - LD3 | 1.133 (0.446,1.82) | 0.0389 |
|  | LD1 - LD4 | 0.203 (-0.484,0.89) | NS |
|  | LD2 - LD3 | 0.45 (-0.237,1.137) | NS |
|  | LD2 - LD4 | -0.48 (-1.167,0.207) | NS |
|  | LD3 - LD4 | -0.93 (-1.617,-0.243) | 0.1194 |
| Strain = Uninfected, generation = F1 | LD1 - LD2 | -2.333 (-3.02,-1.646) | <.0001 |
|  | LD1 - LD3 | 0.283 (-0.404,0.97) | NS |
|  | LD1 - LD4 | -0.807 (-1.494,-0.12) | NS |
|  | LD2 - LD3 | 2.617 (1.93,3.304) | <.0001 |
|  | LD2 - LD4 | 1.527 (0.84,2.214) | 0.0028 |
|  | LD3 - LD4 | -1.09 (-1.777,-0.403) | 0.0502 |
| Strain = wAlbB, generation = F1 | LD1 - LD2 | -0.687 (-1.374,0) | NS |
|  | LD1 - LD3 | 0.047 (-0.64,0.734) | NS |
|  | LD1 - LD4 | -0.82 (-1.507,-0.133) | NS |
|  | LD2 - LD3 | 0.733 (0.046,1.42) | NS |
|  | LD2 - LD4 | -0.133 (-0.82,0.554) | NS |
|  | LD3 - LD4 | -0.867 (-1.554,-0.18) | NS |
| Strain = wMel, generation = F1 | LD1 - LD3 | -0.737 (-1.424,-0.05) | NS |
|  | LD1 - LD4 | -1.237 (-1.924,-0.55) | 0.0206 |
|  | LD3 - LD4 | -0.5 (-1.187,0.187) | NS |

**^* NS - Non-significant p-value^**
