# Supplementary material for: Larval diet-mediated morphological trait and Wolbachia density variation in Wolbachia-transinfected (wMel and wAlbB) and Wolbachia-free Aedes aegypti mosquitoes
Source: Front Insect Sci. 2026 Sep 3;6:1887426. doi: 10.3389/finsc.2026.1887426 (PMC13582537; doi:10.3389/finsc.2026.1887426)
Supplement: Supplementary file 2 [file Table2.docx]

**Supplementary Table 2. Comparison of various competing models involving the three factors Diet, strain and generation**

| **Outcome** | **Model** | **Fixed Effect** | **F Value** | **p-value** | **AIC, R^2^** | **Preferred model** |
| --- | --- | --- | --- | --- | --- | --- |
|  | **Wing length (Male)** | | | |  |  |
| Model 1 | WLM ~ Diet | Diet | 12.45 | <0.001 | 760.7, 0.31 |  |
| Model 2 | WLM ~ Strain | Strain | 0.25 | 0.7824 | 789.6, 0 |  |
| Model 3 | WLM ~ Generation | Generation | 0.16 | 0.6926 | 787.9, 0 |  |
| Model 4 | WLM ~ Diet + strain+ Diet * strain | Diet | 13.64 | <0.001 | 765.0, 0.36 |  |
|  |  | Strain | 1.07 | 0.3514 |  |  |
|  |  | Diet*strain | 1.53 | 0.1859 |  |  |
| Model 5 | WLM~ Diet + generation + Diet * generation | Diet | 14.11 | <0.001 | 751.5, 0.45 | Yes |
|  |  | Generation | 0.12 | 0.7260 |  |  |
|  |  | Diet*Generation | 5.58 | 0.0019 |  |  |
| Model 6 | WLM ~ strain + generation + strain * generation | Strain | 0.19 | 0.8253 | 794.1, 0.01 |  |
|  |  | Generation | 0.13 | 0.7159 |  |  |
|  |  | Strain*Generation | 0.55 | 0.5775 |  |  |
| Model 7 | WLM ~ Diet + Strain + generation+ Diet * Strain + Strain* generation+ generation* Diet | Diet | 14.14 | <0.001 | 756.9, 0 |  |
|  |  | Strain | 0.09 | 0.9181 |  |  |
|  |  | Generation | 0.04 | 0.8439 |  |  |
|  |  | Diet *Strain | 1.41 | 0.2286 |  |  |
|  |  | Diet * Generation | 5.15 | 0.0034 |  |  |
|  |  | Strain* Generation | 1.49 | 0.2359 |  |  |
| Model 8 | WLM ~ Diet +Strain+ generation+ Diet * Strain + Strain * generation+ generation* Diet+ Generation* Diet*strain | Diet | 13.87 | <0.001 | 763.79,0.43 |  |
|  |  | Strain | 0.14 | 0.8701 |  |  |
|  |  | generation | 0.28 | 0.6012 |  |  |
|  |  | Diet *Strain | 1.33 | 0.2639 |  |  |
|  |  | Strain* Generation | 1.40 | 0.2570 |  |  |
|  |  | Generation* Diet | 4.85 | 0.0051 |  |  |
|  |  | Generation* Diet*strain | 0.41 | 0.8426 |  |  |
|  | **Wing Length Female** | | | | | |
| Model 1 | WLF ~ Diet | Diet | 36.64 | <0.001 | 793.2,0.61 |  |
| Model 2 | WLF ~ Strain | Strain | 0.21 | 0.8102 | 859.8, 0 |  |
| Model 3 | WLF ~ Generation | Generation | 11.03 | 0.0015 | 847.07, 0.13 |  |
| Model 4 | WLF ~ Diet + strain+ Diet * strain | Diet | 38.84 | <0.001 | 797.4, 0.63 |  |
|  |  | Strain | 0.24 | 0.7871 |  |  |
|  |  | Diet*strain | 1.72 | 0.1333 |  |  |
| Model 5 | WLF ~ Diet + generation + Diet * generation | Diet | 55.91 | <0.001 | 760.3, 0.77 |  |
|  |  | Generation | 37.37 | <0.001 |  |  |
|  |  | Diet*Generation | 5.32 | 0.0025 |  |  |
| Model 6 | WLF ~ strain + generation + strain * generation | Strain | 0.4 | 0.6752 | 853.4667, 0.09 |  |
|  |  | Generation | 10.93 | 0.0016 |  |  |
|  |  | Strain*Generation | 0.36 | 0.6991 |  |  |
| Model 7 | WLF ~ Diet + Strain + generation+ Diet * Strain + Strain * generation+ generation* Diet | Diet | 59.23 | <0.001 | 758.0, 0.80 |  |
|  |  | Strain | 0.98 | 0.3823 |  |  |
|  |  | Generation | 40.82 | <0.001 |  |  |
|  |  | Diet *Strain | 2.36 | 0.0434 |  |  |
|  |  | Diet * Generation | 5.51 | 0.0023 |  |  |
|  |  | Strain* Generation | 1.57 | 0.2183 |  |  |
| Model 8 | WLF ~ Diet +Strain+ generation+ Diet * Strain + Strain * generation+ generation* Diet+ Generation* Diet*strain | Diet | 82.75 | <0.001 | 743.2, 0.84 | Yes |
|  |  | Strain | 1.33 | 0.2732 |  |  |
|  |  | generation | 50.62 | <0.001 |  |  |
|  |  | Diet *Strain | 3.05 | 0.0135 |  |  |
|  |  | Strain* Generation | 2.03 | 0.1434 |  |  |
|  |  | Generation* Diet | 7.12 | <0.001 |  |  |
|  |  | Generation* Diet*strain | 3.98 | 0.0044 |  |  |
|  | **Body Weight Male** | | | | | |
| Model 1 | BWM ~ Diet | Diet | 4.99 | 0.0036 | 105.29, 0.15 |  |
| Model 2 | BWM ~ Strain | Strain | 5.77 | 0.0049 | 106.46, 0.12 |  |
| Model 3 | BWM ~ Generation | Generation | 2.05 | 0.1570 | 113.5, 0.02 |  |
| Model 4 | BWM ~ Diet + strain+ Diet * strain | Diet | 5.45 | 0.0023 | 78.977, 0.48 |  |
|  |  | Strain | 8.25 | <0.001 |  |  |
|  |  | Diet*strain | 5.51 | <0.001 |  |  |
| Model 5 | BWM ~ Diet + generation + Diet * generation | Diet | 4.79 | 0.0046 | 107.58, 0.17 |  |
|  |  | Generation | 1.48 | 0.2287 |  |  |
|  |  | Diet*Generation | 1.23 | 0.3050 |  |  |
| Model 6 | BWM ~ strain + generation + strain * generation | Strain | 6.39 | 0.0030 | 100.98, 0.22 |  |
|  |  | Generation | 3.01 | 0.0879 |  |  |
|  |  | Strain*Generation | 4.02 | 0.0227 |  |  |
| Model 7 | BWM ~ Diet + Strain + generation+ Diet * Strain + Strain * generation+ generation* Diet | Diet | 7.02 | <0.001 | 55.4, 0.65 |  |
|  |  | Strain | 10.97 | <0.001 |  |  |
|  |  | Generation | 5.05 | 0.0289 |  |  |
|  |  | Diet *Strain | 8.15 | <0.001 |  |  |
|  |  | Diet * Generation | 2.91 | 0.0431 |  |  |
|  |  | Strain* Generation | 9.14 | <0.001 |  |  |
| Model 8 | BWM ~ Diet +Strain+ generation+ Diet * Strain + Strain * generation+ generation* Diet+ Generation* Diet*strain | Diet | 16.15 | <0.001 | 18.556, 0.80 | Yes |
|  |  | Strain | 20.43 | <0.001 |  |  |
|  |  | generation | 9.89 | 0.0029 |  |  |
|  |  | Diet *Strain | 14.5 | <0.001 |  |  |
|  |  | Strain* Generation | 16.25 | <0.001 |  |  |
|  |  | Generation* Diet | 5.18 | 0.0036 |  |  |
|  |  | Generation* Diet*strain | 8.95 | <0.001 |  |  |
|  | **Body Weight Female** | | | | | |
| Model 1 | BWF ~ Diet | Diet | 2.27 | 0.082 | 165.56, 0.05 |  |
| Model 2 | BWF ~ Strain | Strain | 2.27 | 0.113 | 165.855, 0.04 |  |
| Model 3 | BWF ~ Generation | Generation | 0.19 | 0.6665 | 168.25,0 |  |
| Model 4 | BWF ~ Diet + strain+ Diet * strain | Diet | 2.33 | 0.0836 | 166.41, 0.13 |  |
|  |  | Strain | 2.73 | 0.0735 |  |  |
|  |  | Diet*strain | 1.54 | 0.1826 |  |  |
| Model 5 | BWF ~ Diet + generation + Diet * generation | Diet | 4.19 | 0.0093 | 139.9, 0.3803 |  |
|  |  | Generation | 1.25 | 0.2682 |  |  |
|  |  | Diet*Generation | 12.64 | <0.001 |  |  |
| Model 6 | BWF ~ strain + generation + strain * generation | Strain | 2.16 | 0.1233 | 167.07, 0.0577 |  |
|  |  | Generation | 0.13 | 0.7235 |  |  |
|  |  | Strain*Generation | 2.21 | 0.1182 |  |  |
| Model 7 | BWF ~ Diet + Strain + generation+ Diet * Strain + Strain * generation+ generation* Diet | Diet | 6.57 | <0.001 | 122.96, 0.57 |  |
|  |  | Strain | 1.37 | 0.2641 |  |  |
|  |  | Generation | 2.84 | 0.0983 |  |  |
|  |  | Diet *Strain | 3.09 | 0.0118 |  |  |
|  |  | Diet * Generation | 17.83 | <0.001 |  |  |
|  |  | Strain* Generation | 7.01 | 0.0020 |  |  |
| Model 8 | BWF ~ Diet +Strain+ generation+ Diet * Strain + Strain * generation+ generation* Diet+ Generation* Diet*strain | Diet | 6.31 | <0.001 | 118.49, 0.61 | Yes |
|  |  | Strain | 2.06 | 0.1388 |  |  |
|  |  | generation | 1.58 | 0.2145 |  |  |
|  |  | Diet *Strain | 3.43 | 0.0069 |  |  |
|  |  | Strain* Generation | 7.79 | 0.0012 |  |  |
|  |  | Generation* Diet | 19.84 | <0.001 |  |  |
|  |  | Generation* Diet*strain | 2.15 | 0.0767 |  |  |
