# Supplementary material for: Larval diet-mediated morphological trait and Wolbachia density variation in Wolbachia-transinfected (wMel and wAlbB) and Wolbachia-free Aedes aegypti mosquitoes
Source: Front Insect Sci. 2026 Sep 3;6:1887426. doi: 10.3389/finsc.2026.1887426 (PMC13582537; doi:10.3389/finsc.2026.1887426)
Supplement: Supplementary file 3 [file Table3.docx]

**S3. Details of final primer and probe concentrations used in each reaction for detecting *Wolbachia* density**

| **Reagent** | **Volume** |
| --- | --- |
| 2x LightCycler 480 Probe Master (Roche, Switzerland) | 5 µL |
| 10 µM forward primer 1 (wspTM2 or Alb_16009) | 0.25 µL |
| 10 µM reverse primer 1 (wspTM2 or Alb_16009) | 0.25 µL |
| 10 µM probe 1  (wspTM2 or Alb_16009) | 0.10 µL |
| 10 µM forward primer 2 (Rps17) | 0.25 µL |
| 10 µM reverse primer 2 (Rps17) | 0.25 µL |
| 10 µM probe 2  (Rps17) | 0.10 µL |
| Nuclease-free water | 2.80 µL |
| DNA template | 1 µL |
